# Calcium/calmodulin-dependent protein kinases in the carotid body: an immunohistochemical study

**DOI:** 10.1186/2193-1801-1-16

**Published:** 2012-08-27

**Authors:** Mieczysław Pokorski, Hiroyuki Sakagami, Yasumasa Okada

**Affiliations:** 1 Department of Respiratory Research, Medical Research Center, Polish Academy of Sciences, Warsaw, and Department of Neuropsychology, Institute of Psychology, Opole University, Opole, Poland; 2 Laboratory of Electrophysiology, Clinical Research Center, Murayama Medical Center, MusashiMurayama, Tokyo, Japan; 3 Department of Anatomy, Kitasato University School of Medicine, Sagamihara, Kanagawa, Japan

**Keywords:** Ca^2+^/calmodulin-dependent protein kinase, Carotid body, Chemoreceptor cells

## Abstract

We determined the presence of Ca^2+^/calmodulin-dependent protein kinases (CaMKs), a family of multifunctional proteins engaged in Ca^2+^-linked signaling, in carotid body chemoreceptor cells which are critical for the hypoxia-sensing. Carotid bodies were dissected from anesthetized normoxic adult Wistar rats and were double stained for individual CaMKs and for tyrosine hydroxylase (TH), a marker of chemoreceptor cells. Immunofluorescence was examined by confocal laser scanning microscopy. We found that CaMKI and CaMKII were expressed in chemoreceptor cells, but their distribution and intensity varied. CaMKI immunoreactivity was distributed throughout the cytoplasm, whereas that of CaMKII was localized in the cytoplasmic periphery of chemoreceptor cells. An overlap of CaMKI or CaMKII fluorescent probes with TH affirmed the presence of either protein in the chemoreceptor cells. CaMKIV could not be conclusively visualized by the used method. The study shows the expressions of CaMKI and CaMKII in chemoreceptor cells, which raises the plausibility of CaMKs` role in carotid body function.

## Background

Ca^2+^/calmodulin (CaM)-dependent protein kinases (CaMKs): CaMKI, CaMKII, and CaMKIV are a family of proteins which, through phosphorylation of intracellular substrates, mediate a host of neural functions, including neurotransmitter metabolism, long-term facilitation, and gene transcription. CaMKs are foremost the key regulators of cellular responses to Ca^2+^/calmodulin mobilizing stimuli (Hook & Means [2001]).

Hypoxic hypoxia unleashes pulmonary hyperventilation, a primary reflex defense reaction, aimed at sustaining delivery of O_2_ to tissues. The reaction is explicitly generated by the carotid body chemoreceptor cells which detect the deficit of O_2_ and transduce that extracellular signal into the intracellular Ca^2+^ rise (Pokorski et al. [2012]), followed by neurotransmitter release and a signaling cascade leading eventually to cell excitation and increased neural discharge rate from the organ (Di Giulio et al. [2012]; Zara et al. [2012]). Ca^2+^ transients are indubitable for the chemoreceptor responses, as these responses are absent after Ca^2+^ chelation (Obeso et al. [1992]). CaMKs, fundamentally important proteins for translating transiently evoked Ca^2+^ signals into sustained cellular responses (Hudmon & Schulman [2002]), seem well suited to be germane to carotid body function. Indeed, immunoreactivity for CaMKI has been found in the glomus cells, although the exact intracellular location of the enzyme is not well defined (Hoshi et al. [2006]). The presence of other CaMKs has never been substantiated in carotid chemoreceptor cells. Therefore, in the present study we endeavored to delineate the expression of individual CaMKs in the normoxic carotid chemoreceptor cells by immunocytochemistry.

## Results

**Figure 1:**
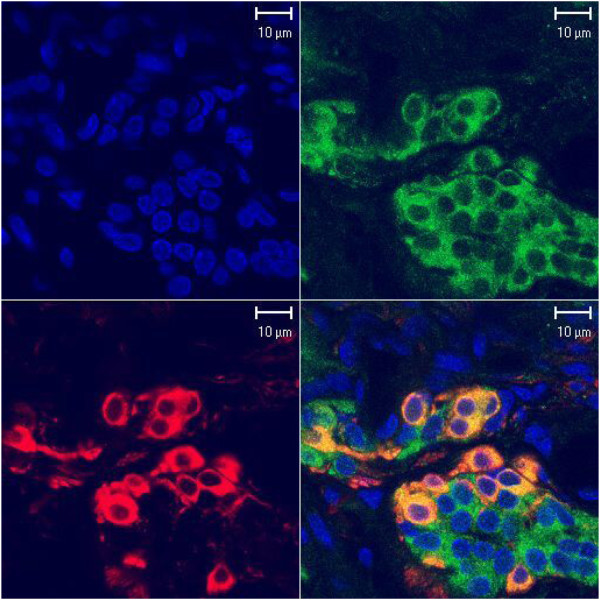
**Confocal laser scanning microscopic images of CaMKI immunostaining in the rat carotid body.** CaMKI immunostaining in clusters of chemoreceptor cells of the rat carotid body. Consecutive panels show, from left, nuclear staining (blue), anti-CaMKI (green), anti-TH (red), and the overlay of the two fluorescence probes (yellow). CaMKI immunoreactivity overlaps with that of TH in chemoreceptor cells.

**Figure 2:**
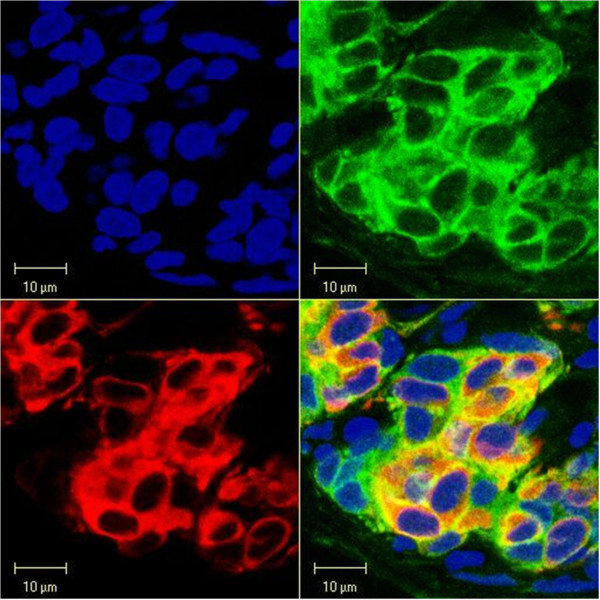
**CaMKII immunoprecipitates in the rat carotid body.Localization of CaMKII immunoprecipitates in chemoreceptor cells of the rat carotid body.** Consecutive panels show, from left, nuclear staining (blue), anti-CaMKI (green), anti-TH (red), and the overlay of the two fluorescence probes (yellow). CaMKII immunoreactivity overlaps with that of TH in chemoreceptor cells.

**Figure 3:**
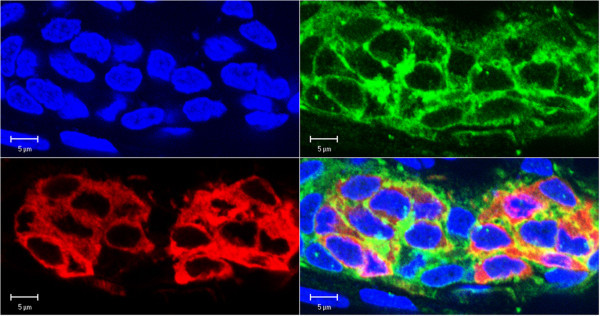
**Overlap of CaMKII and TH immunoprecipitates. An example of the overlap of CaMKII and TH immunoprecipitates of varied density in neighboring clusters of chemoreceptor cells in the rat carotid body.** Consecutive panels show, from left, nuclear staining (blue), anti-CaMKI (green), anti-TH (red), and the overlay of the two fluorescence probes (yellow).

We found that CaMKI and CaMKII, but not CaMKIV, were expressed in carotid chemoreceptor cells. Representative examples of the expression pattern of CaMKI are displayed in Figure 1 and of CaMKII in Figures 2 & 3. The successive panels in all figures show nuclear staining (blue), anti-CaMK staining (green), anti-TH staining (red), and an overlay of the latter two (yellow). Each figure shows clusters of chemoreceptor cells, as identified by the TH marker staining, of 10–15 μm in diameter with irregularly oval nuclei. The amount and intensity of both TH and CaMK immunostaining varied between individual cells and clusters of cells, depending on the sectioning of cells. There were differences in the distribution pattern of the two CaMKs, as assessed by visual inspection, based on the density of immunostaining.

The immunostaining of CaMKI had a fine appearance and was distributed throughout the cytoplasm of the chemoreceptor cells, as evidenced by an overlap with the localization of TH expression (Figure 1). There were occasional isles of cells with a degree of mismatch of immunostaining for CaMKI and TH. The mismatch plausibly concerns the cells surrounding a microvessel (Figure 1 - center of the bottom cell cluster), although the method used could not ascertain the exact morphological location. CaMKII, on the other hand, was expressed more intensely in the peripheral layer of the perikaryal cytoplasm, leaving an unstained band encircling the nucleus. The nuclei themselves were devoid of enzyme’s expression. The appearance of immunoprecipitates was consistently more granular than that of CaMKI. Double staining for CaMKII and TH showed co-localization of both fluorescent probes. A dovetailed overlap affirms the presence of the protein in the chemoreceptor cells (Figure 2). There were intra-cluster and inter-cluster differences in the intensity of immunostainings (Figure 3). However, the CaMKII immunostaining closely matched that of TH. The expression pattern of CaMKs was highly reproducible in the examined specimens, sampled from carotid bodies of various rats.

CaMKIV could not be conclusively substantiated in chemoreceptor cells. Rather weak and erratic expression of it was seen in the cytoplasm of the cells positive for TH staining and in the nuclei of the cells negative for TH staining. No other fragments of carotid body parenchyma, such as sustentacular cells or nerve endings, were positively identified as expressing any of the CaMKs studied.

## Discussion

The present study was designed to identify the presence of individual CaMK proteins in carotid body parenchyma. This purpose stemmed from the premise that for an enzyme to have a possible functional meaning it should be present in the organ in question in the first place. Here we report the presence of CaMKI and CaMKII, but not that of CaMKIV, in the rat chemoreceptor cells. Our study expands on the previous finding of CaMKI expression in the carotid body (Hoshi et al. [2006]) in that it is the first visualization of the distribution of individual CaMKs in chemoreceptor cells in the basal, non-stimulated condition.

CaMKII has previously been implicated in hypoxic signaling pathways in PC12 rat pheochromocytoma cells. CaMKII apparently mediates hypoxic activation of *c-fos* gene expression (Premkumar et al. [2000]) and it also is influential in inducing the hypoxia-inducible factor-1α (HIF-1α) transcriptional activity through Ca^2+^-mediated gene regulation in this cell line during intermittent hypoxia (Yuan et al. [2005]). By contrast, the CaMKII signaling pathway seems to have no major bearing on the HIF-1α induction in the PC12 cells exposed to sustained hypoxia (Premkumar et al. [2000]), which is due to a decreased rate of O_2_-dependent proline hydroxylation and, consequently, decreased HIF-1α degradation (Kline et al. [2002]).

The PC12 cells share some neurotransmitter composition with the carotid chemoreceptor cells, but that is not necessarily tantamount to their emulating the chemosensing process of the carotid body. The present results give a consistent impression that CaMKII was expressed strongly in chemoreceptor cells. CaMKII encompasses a family of 28 isoforms that are derived from 4 genes and are stratified into α, β, γ, and δ subunits (Hudmon and Schulman [2002]). The first two predominate in the brain. It is unclear which subunits predominate in the carotid body and our immunohistochemical study would not discern that. CaMKI is distinct from CaMKII in that its major alpha isoform is localized predominantly to the cytoplasm and is a candidate CaMK for the regulation of gene transcription *in vivo* (Picciotto et al. [1995]).

Since both HIF-1α (Lahiri et al. [2007]) and hypoxia (Peng et al. [2004]) affect O_2_-linked adaptable processes in the carotid body and, on the other side, CaMKII is considered a molecular memory enzyme that underlines synaptic plasticity (Fink and Meyer [2002]), its potential role in carotid body function raises a research interest. CaMKII is autophosphorylated in a Ca^2+^/calmodulin-dependent manner, after which it converts to a Ca^2+^-independent form (Miller and Kennedy [1986]). The process prolongs the activity of CaMKII beyond the restoration of a pre-stimulus Ca^2+^ level (Hudmon and Schulman [2002]). That, physiologically, translates into the continuation of a stimulus-induced change after the stimulus discontinuation, a phenomenon known in respiratory responses (Pokorski and Serebrovskaya [2009]). CaMKII could thus shape the carotid chemoafferent plasticity, the molecular basis of which is not well understood.

The experimental design employed in the current study was but capable of showing the expression of total constitutive CaMKs and did not identify their isoforms which may differ in subcellular distribution and function. Furthermore, the static non-stimulated content of CaMKs is shown, whereas CaMKI regulatory function is usually linked to the stimulus-induced translocation to the postsynaptic densities (Strack and Colbran [1998]). Such densities between the abutting chemoreceptor cells or the chemoreceptor cells and sinus nerve endings are the sites of interaction of neurotransmitters, released from secretory vesicles during chemoexcitation, with their specific receptors (Di Giulio et al. [2009]). An increase in chemoreceptor cell cytoplasmic Ca^2+^ during instantaneous responses to acute hypoxia (Pokorski et al. [2012]) is liable to activate CaMKs. However, it is unknown of how exactly the CaMK signaling coalesces with the hypoxia-sensing cascade in the carotid body.

## Conclusions

We herein report the highly reproducible distribution patterns of the CaMKI and CaMKII expressions in carotid chemoreceptor cells. The inference is that CaMK signaling pathways might participate in shaping the chemosensory responses. Confirmation of this biological plausibility requires further studies.

## Methods

The study was carried out on tissues dissected from 4 adult, 1 male and 3 female, Wistar rats (weight 140–270 g; age 7–16 wk), handled in accord with the Guiding Principles for Care and Use of Animals of the Physiological Society of Japan. The rats were deeply anesthetized with diethyl ether, and after opening the chest were instantly perfused through the heart with 4% paraformaldehyde in phosphate buffered saline (PBS), pH 7.20. The carotid bodies were dissected bilaterally from the carotid bifurcation area. The nearby sympathetic superior cervical ganglions also were dissected to be used as reference specimens for both CaMKs and tyrosine hydroxylase (TH), a marker of chemoreceptor cells. Additionally, the brain was extracted from the skull and the hippocampus was cut out for positive CaMKs controls. All the tissues were immersed in the same fixative in which they were postfixed for 1.5 h and then cryoprotected in 30% sucrose at 4°C until further use.

Sections of the carotid body (10 μm thick), superior cervical ganglion (15 μm thick), and hippocampal (30 μm thick) tissues were made (CM1900 cryostat, Leica Instruments, Nussloch, Germany) and attached to glass slides coated with poly-L-lysine. Sections from all the tissues were processed on the same glass slide for a given CaMK.

The immunohistochemical procedures were performed in room temperature and consisted of five successive steps. I – solubilization of specimens in 0.3% Triton X-100 in PBS for 15 min; II – blocking the nonspecific binding with 5% normal goat serum for 30 min; III – overnight incubation with primary rabbit IgG rabbit antibodies against CaMKI, CaMKII, and CaMKIV (dilution 1:1000, 1:1000, and 1 3 μg/ml, respectively) and with a PCTH-7 mouse monoclonal IgG antibody against TH for double staining to confirm the chemoreceptor cell localization of immunostaining; IV – 1 h incubation with secondary goat Alexa Fluor 488-conjugated antirabbit + goat Alexa Fluor 594-conjugated antimouse antibodies diluted at 1:2000 (Alexa Flour; Molecular Probes, Eugene, OR) in the environment protected from light; and V – nuclear staining with 4',6-diamidino-2-phenylindole (DAPI; Sigma-Aldrich, St. Louis, MO). Steps III, IV, and V were followed by several sequential washes of specimens in PBS. Finally, the specimens were covered with an embedding medium and examined by visual inspection and photographed under a confocal laser scanning microscope and workstation (LSM 5 Pascal; Carl Zeiss, Jena, Germany).

Antibodies against CaMKI were produced in-house. Briefly, the entire coding region of rat CaMKIα was amplified by PCR and inserted in frame to pGEX4T-1 expression vector (Amersham Biosciences, Piscataway, NJ). The glutathione S-transferase (GST)-CaMKIα fusion protein was induced in *E. coli* in the presence of isopropyl-1-thio-β-D-galactoside. The fusion protein was then purified from a soluble extract by glutathione-Sepharose 4B (Amersham Biosciences, Piscataway, NJ) according to the manufacturer’s protocol. Two female New Zealand rabbits were immunized by repeated intradermal injections of the fusion protein emulsified with an equal volume of Freund's adjuvant. The antibody against CaMKI was purified by a protein A-sepharose 4B column, followed by the A-sepharose 4B column coupled with GST-CaMKIα. Western blots showed that the antibody reacted with CaMKIα and δ, but not β2 and γ1 in a single band. Antibodies against CaMKIV were made according to the method described previously (Sakagami et al. [1994]) and those against CaMKII were generously provided by Prof. Kohji Fukunaga of Tohoku University Graduate School of Pharmaceutical Sciences.

Positive controls showed the CaMKs immunostainings in the hippocampus, which was particularly relevant regarding the CaMKIV that appeared as streaks of stained neuronal nuclei. The CaMKs and TH closely overlapped in the sympathetic neurons of the superior cervical ganglion, as is reported in the literature (Arciszewski et al. [2004]). Negative controls, in which the primary antibody was omitted, failed to express immunostaining. Thus, both positive and negative controls (not shown) verified the soundness of the immunostaining.
